# Narirutin-Rich Celluclast Extract from Mandarin (*Citrus unshiu*) Peel Alleviates High-Fat Diet-Induced Obesity and Promotes Energy Metabolism in C57BL/6 Mice

**DOI:** 10.3390/ijms25084475

**Published:** 2024-04-19

**Authors:** Seung Tae Im, Heejoo Kang, Jusang Kim, Song-Rae Kim, Kil-Nam Kim, Seung-Hong Lee

**Affiliations:** 1 Department of Medical Science, Soonchunhyang University, Asan 31538, Republic of Korea; lama1010@naver.com; 2 ILHAE Co., Ltd., Jeju 695962, Republic of Korea; hot_hwi@naver.com (H.K.); jusangi@ilhae.co.kr (J.K.); 3 Metropolitan Seoul Center, Korea Basic Science Institute, Seoul 02841, Republic of Korea; ksr87@kbsi.re.kr; 4 Gwangju Center, Korea Basic Science Institute, Gwangju 61751, Republic of Korea; knkim@kbsi.re.kr; 5 Department of Pharmaceutical Engineering, Soonchunhyang University, Asan 31538, Republic of Korea

**Keywords:** mandarin peel, enzymatic extract, HFD-induced obese mice, anti-obesity effect, functional food

## Abstract

Mandarin peel, a main by-product from the processing of citrus juice, has been highlighted for its various bioactivities and functional ingredients. Our previous study proved the inhibitory effects of Celluclast extract from mandarin peel (MPCE) on lipid accumulation and differentiation in 3T3-L1 adipocytes. Therefore, the current study aimed to evaluate the anti-obesity effect of MPCE in high-fat diet (HFD)-induced obese mice. The high-performance liquid chromatography (HPLC) analysis exhibited that narirutin and hesperidin are the main active components of MPCE. Our current results showed that MPCE supplementation decreased adiposity by reducing body and organ weights in HFD-induced obese mice. MPCE also reduced triglyceride (TG), alanine transaminase (ALT), aspartate transaminase (AST), and leptin contents in the serum of HFD-fed mice. Moreover, MPCE significantly inhibited hepatic lipid accumulation by regulating the expression levels of proteins associated with lipid metabolism, including sterol regulatory element-binding protein (SREBP1c), fatty acid synthase (FAS), and acetyl-CoA carboxylase (ACC). Furthermore, MPCE administration significantly inhibited both adipogenesis and lipogenesis, with modulation of energy metabolism by activating 5′ adenosine monophosphate-activated protein kinase (AMPK) and lipolytic enzymes such as hormone-sensitive lipase (HSL) in the white adipose tissue (WAT). Altogether, our findings indicate that MPCE improves HFD-induced obesity and can be used as a curative agent in pharmaceuticals and nutraceuticals to alleviate obesity and related disorders.

## 1. Introduction

Obesity is a representative broad health concern that is characterized by an excess body weight increase, which is associated with the prevalence of various metabolic diseases, such as diabetes, dyslipidemia, hypertension, and cardiovascular diseases [1]. It is defined as a major chronic disease and is involved in the risk of increasing global mortality. The progression and severity of obesity are closely related to multiple factors that include environmental and genetic factors; however, the fundamental cause is an energy imbalance between calory intake and consumption due to the deterioration of dietary behavior and physical activity [2,3].

The most common treatment for obesity is weight loss through the regulation of food intake and the maximization of energy expenditure. For obese patients, many limits interfere with these lifestyle changes, suggesting the use of a pharmacological agent that contributes to weight regulation [4,5]. These anti-obesity drugs, such as orlistat, rimonabant, and sibutramine, result in weight loss by regulating appetite and nutrition metabolism. Although their treatment benefits are effective, the risk of side effects and adverse toxicities may outweigh their usefulness [4,6]. Therefore, therapeutic strategies utilizing natural compounds have attracted more attention due to their advantages, which include safety and efficiency [3,6].

Citrus fruit, one of the most abundant fruit crops worldwide, is rich in beneficial phytochemicals, such as phenolic compounds, vitamins, and dietary fiber [7,8]. It is well known that citrus fruit and its components contribute to multiple bioactive properties, including antioxidant, anti-inflammatory, anti-tumor, anti-diabetic, and even anti-obesity [7,9,10]. Additionally, the colored outer layer of the rind of citrus fruit that is not edible, the citrus peel, is also responsible for the various health benefits mentioned above and is a promising source of natural flavonoids [11,12]. However, a large amount of citrus peel, which is produced as a by-product during citrus juice processing, is still regarded as a primary waste and implicated in economic and environmental issues [8,11]. In this context, there is increasing interest in utilizing citrus peel as a pharmaceutical and functional ingredient for potential human health properties [8].

Flavanones are natural phytochemicals that are highly present in citrus peel and that have multifunctional bioactivities [13]. In particular, the main members of the flavanone group of flavonoids, such as hesperidin and narirutin, have revealed their potential in the treatment of obesity and its metabolic complications, like type 2 diabetes, inflammatory response, and hypertension [14,15,16,17]. Against this backdrop, it is necessary to develop an optimized extraction method to prepare these bioactive compounds from citrus peel that can be used in the development of nutraceutical and pharmaceutical ingredients [18].

Traditionally, the main formulations of citrus peel are obtained by extraction with hot water or organic solvents, which have been used for a long time [19]. However, this approach has some disadvantages, such as restrictive usage in the food industry, low extraction yield, and physiological activity. Therefore, a safe and effective extraction method is required to obtain acceptable bioactive ingredients as functional foods and nutraceuticals from citrus peel by-products. In recent years, enzymatic extraction has commonly been applied for the successful extraction of numerous bioactive ingredients with biological properties from a wide variety of fruit and industrial crops [20,21]. This technique improves the extraction yields of targeted bioactive components, and the extracted components often exhibit enhanced bioactivities as well as less toxicity compared with classical extraction methods [21,22]. On one hand, our previous study showed that Celluclast extract from mandarin peel (MPCE) significantly inhibited pre-adipocyte differentiation and lipid accumulation in 3T3-L1 cells [23].

However, to the best of our knowledge, the in vivo anti-obesity effect and bioactive components of MPCE have not yet been fully investigated. To further investigate the value of both industrial utilization and potential functional food ingredients of MPCE, the present study examines the effect of MPCE on high-fat diet (HFD)-induced obese mice and the analysis of bioactive components.

## 2. Results

### 2.1. Flavonoid Composition of MPCE

**Figure 1:**
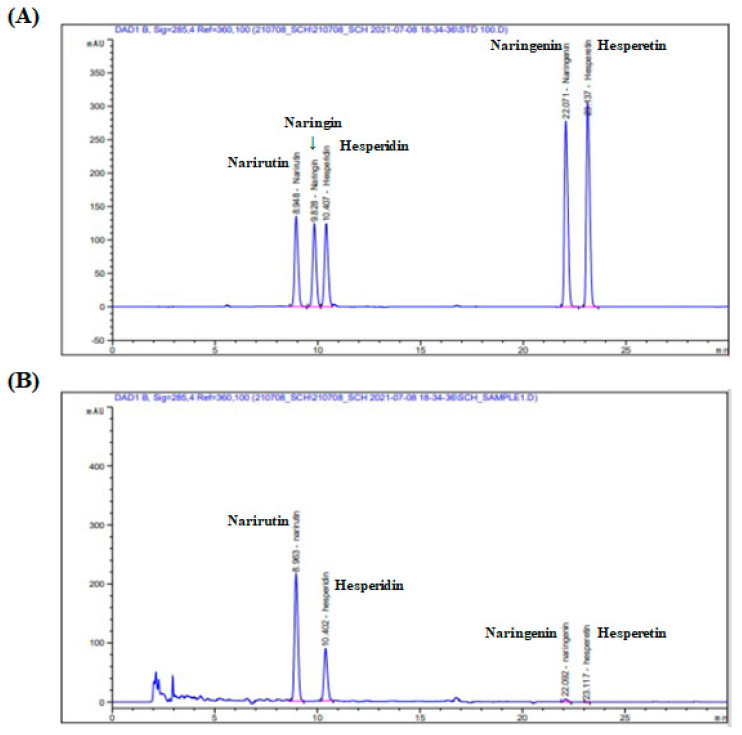
The chromatograms of (**A**) five flavanone standards and (**B**) mandarin peel Celluclast extract (MPCE).

To our knowledge, mandarin peel contains abundant flavonoids. Therefore, the flavonoid composition of MPCE was carried out based on the literature data and available reference standards by using HPLC analysis [8,24]. Figure 1 shows the flavonoid composition of MPCE. Five flavonoid standards (narirutin, naringin, hesperidin, naringenin, and hesperetin) were used to determine the flavonoid content of MPCE. Out of the five standards used, three flavonoids were detected and quantified in MPCE. Narirutin was indicated as the major compound based on its high quantity (13.795 mg/g), followed by hesperidin (6.597 mg/g) and naringenin (0.167 mg/g). These results suggest that the major flavonoid component of MPCE is narirutin and might have contributed to the anti-obesity effects of MPCE.

### 2.2. Effects of MPCE on HFD-Induced Adiposity in Obese Mice

**Figure 2:**
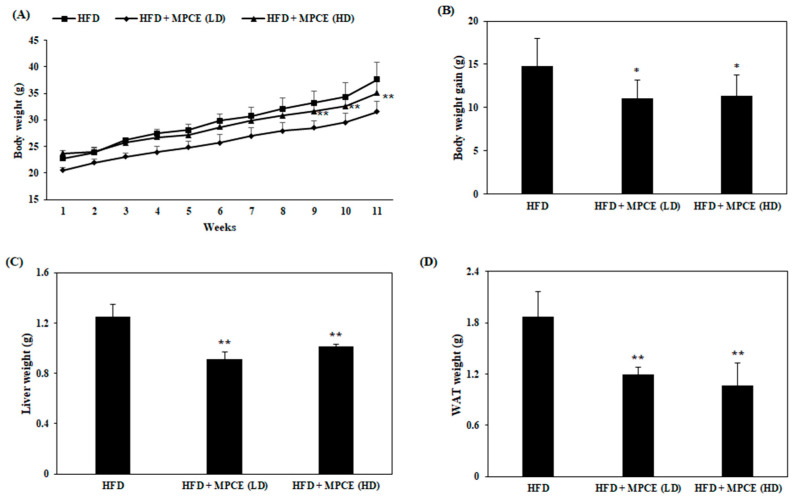
MPCE decreases adiposity in high-fat diet (HFD)-induced obese mice. (**A**) The bodyweight (**B**) and the body weight gain of the mice measured for 11 weeks. The weights of (**C**) liver and (**D**) white adipose tissue (WAT) of HFD-treated or MPCE co-treated mice. Data are expressed as the mean ± standard deviation (*n* = 6). * *p* < 0.05, ** *p* < 0.01, indicate significant differences compared with the only HFD group.

HFD rodent models have commonly been used to understand obesity in terms of body weight control and physiology [25]. Therefore, we fed mice with HFD or HFD administered with MPCE, and the body weights of mice were measured weekly to investigate the effect of MPCE on adiposity. Figure 2A shows the body weight changes during the experiment. MPCE administration had significantly lower body weight compared with HFD-fed mice. The body weight gain for 11 weeks for the HFD group was 14.83 g, and for the groups with 125 and 200 mg/kg MPCE supplementation, it was 11.08 and 11.35 g, respectively (Figure 2B). Moreover, the liver weights (Figure 2C) and white adipose tissue (WAT) weights (Figure 2D) of mice were significantly reduced by MPCE supplementation. These findings suggest that the MPCE treatment alleviated HFD-induced adiposity.

### 2.3. Effects of MPCE on HFD-Induced Serum Biological Parameters in Obese Mice

Table 1 shows the serum biochemical levels. For 11 weeks, the serum levels of TG and leptin were significantly lower in the MPCE-treated mice groups than in the HFD-only group. In addition, MPCE-treated mice showed significantly lower AST and ALT, which is indicative of liver damage, than the mice fed with HFD only. These results showed that MPCE effectively ameliorated these obesity-related biological parameters in HFD-induced obese mice.

### 2.4. Effects of MPCE on Hepatic Lipid Accumulation and the Expression Levels of Lipid Metabolism-Related Proteins in HFD-Induced Obese Mice

**Figure 3:**
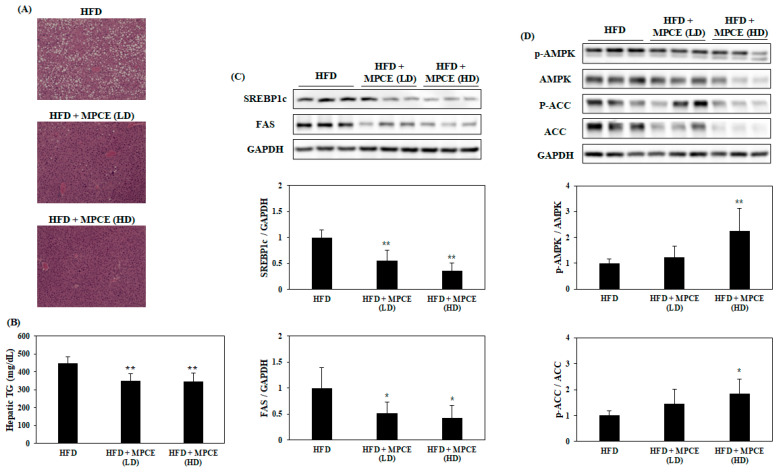
MPCE treatment inhibits HFD-induced hepatic lipid accumulation and modulates the expression of key proteins and enzymes related to lipid metabolism in obese mice. (**A**) Representative images of hematoxylin and eosin (H&E)-stained liver tissues, and (**B**) relative TG levels. (**C**) The expression levels of lipogenesis-related proteins and (**D**) the phosphorylation levels of fatty acid β-oxidation-related proteins were determined by Western blot analysis. Data are expressed as the mean ± standard deviation (*n* = 6). * *p* < 0.05, ** *p* < 0.01, indicate significant differences compared with the only HFD group.

Fatty liver, characterized by excessive hepatic lipid accumulation and dysregulation, is closely implicated with obesity and metabolic disorders [26]. Hence, we investigated the effects of MPCE on hepatic lipid accumulation. Hematoxylin & Eosin (H&E) stains were used to stain liver tissues for the lipid accumulation observation. This observation shows that MPCE administration significantly prevented hepatic lipid accumulation compared with HFD-fed mice (Figure 3A). This result was clearly validated by measuring TG levels in the liver (Figure 3B). To clarify the mechanisms by which MPCE inhibits hepatic lipid accumulation, Western blot analysis was performed to determine the expression levels of key proteins related to hepatic lipid metabolism. As expected, the results showed that the MPCE treatment significantly reduced the expression levels of lipogenic-specific proteins and enzymes, such as sterol regulatory element-binding protein 1 (SREBP1c) and fatty acid synthase (FAS) (Figure 3C). In addition, MPCE treatment significantly increased the phosphorylation levels of 5’ adenosine monophosphate-activated protein kinase (AMPK) and acetyl-CoA carboxylase (ACC) (Figure 3D), promoting fatty acid oxidation and ultimately inhibiting lipid accumulation [27]. Taken together, these results suggest that MPCE effectively prevents lipid accumulation in the liver by downregulating SREBP1c and FAS, based on AMPK and ACC phosphorylation.

### 2.5. Effects of MPCE on HFD-Induced Adipogenesis and Lipogenesis in the WAT of Obese Mice

**Figure 4:**
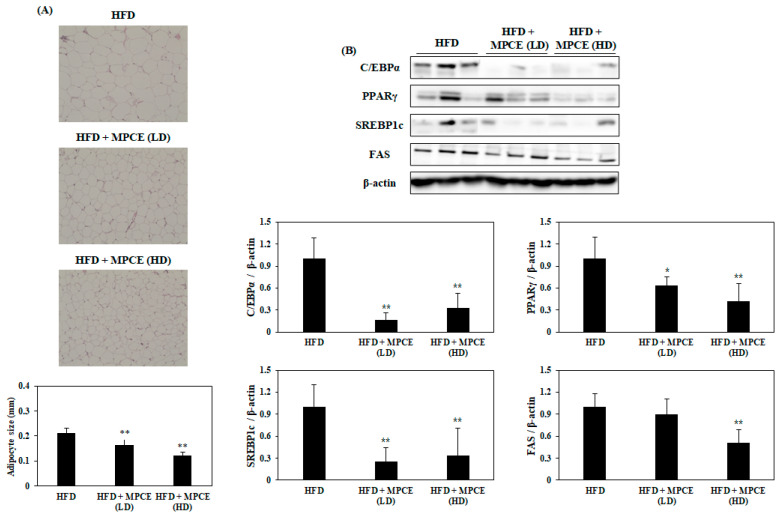
MPCE treatment reduces HFD-induced adipogenesis and lipogenesis in the WAT of obese mice. (**A**) Representative images of H&E-stained WATs. (**B**) The expression levels of adipogenesis and lipid synthesis-related proteins were determined by Western blot analysis. Data are expressed as the mean ± standard deviation (*n* = 6). * *p* < 0.05, ** *p* < 0.01, indicate significant differences compared with the only HFD group.

Figure 4A shows the results of the histological analysis in the WAT, which exhibited that the mice administered with MPCE have smaller adipocyte sizes than the HFD-induced obese mice. To determine the effects of MPCE on adipogenesis and lipogenesis, the expression levels of both adipogenesis- and lipogenesis-related proteins were measured in the WAT. Based on Western blot analysis, MPCE treatment significantly decreased the expression levels of specific proteins related to adipogenesis and lipid synthesis, including peroxisome proliferator-activated receptor gamma (PPARγ), CCAAT/enhancer-binding protein alpha (C/EBPα), SREBP1c, and FAS, in the WAT of HFD-fed obese mice (Figure 4B). Collectively, our results indicate that MPCE reduces adipocyte size by inhibiting the expression levels of key adipogenic-specific proteins and lipogenic enzymes in the WAT of HFD-induced mice.

### 2.6. Effects of MPCE on HFD-Induced Lipolytic Metabolism in the WAT of Obese Mice

**Figure 5:**
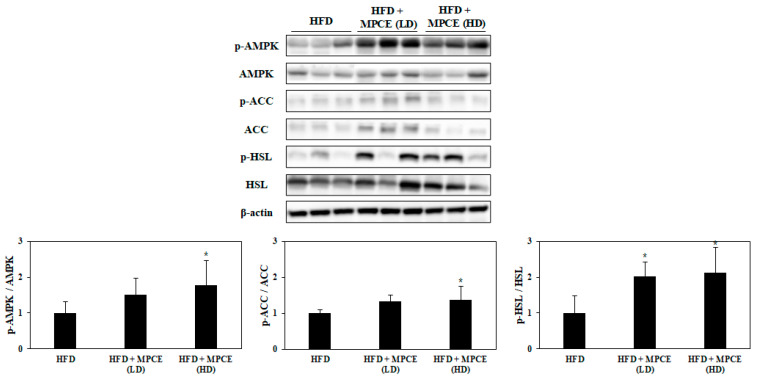
MPCE treatment improves HFD-induced lipolytic energy metabolism in the WAT of obese mice. The phosphorylation levels were determined by Western blot analysis. Data are expressed as the mean ± standard deviation (*n* = 6). * *p* < 0.05 indicates significant differences compared with the only HFD group.

To further investigate other mechanisms associated with the anti-adipogenesis effect of MPCE, we examined the phosphorylation levels of AMPK, ACC, and hormone-sensitive lipase (HSL) in the WAT. Figure 5 shows that the MPCE-treated mice enhanced phosphorylation levels of AMPK and ACC, associated with fatty acid β-oxidation. Moreover, MPCE notably promoted lipolytic enzyme HSL phosphorylation in HFD-induced obese mice (Figure 5). These findings suggest that MPCE could prevent HFD-induced obesity by promoting fatty acid β-oxidation and lipolysis.

## 3. Discussion

Obesity is a global epidemic that is defined by overnutrition and follows an excessive increase in body mass index (BMI). It is well known that obesity contributes to diverse metabolic diseases, like diabetes mellitus, cardiovascular diseases, non-alcoholic fatty liver diseases (NAFLD), and even certain types of cancer [28]. Currently, over one-third of the world’s population is regarded as overweight or obese, and the prevalence of obesity is still increasing [29]. Thus, it is crucial to develop effective agents and therapeutic strategies for the recovery of public health.

There are several synthetic anti-obesity agents, such as orlistat and sibutramine, that interfere with systemic metabolism; however, many studies have reported their unhealthy adverse effects [30,31]. Therefore, the development of natural candidates exhibiting anti-obesity effects has been focused on in the food research area for their lesser toxicity and additional potential health benefits [31]. Recently, many previous reports suggested that the peel of citrus fruits contains bioactive compounds and has the potential to suppress obesity [32,33,34]. Moreover, our previous study results indicate that the Celluclast extract of mandarin (*Citrus unshiu*) peel significantly inhibited intracellular lipid accumulation in 3T3-L1 adipocytes [23]. However, there have been no reports of bioactive components that can be extracted from mandarin peel by Celluclast hydrolysis. In addition, to use the Celluclast extract of mandarin peel (MPCE) as a potential therapeutic candidate against obesity, the underlying mechanisms of the anti-obesity effect of MPCE require further elucidation in an in vivo model. Thus, the objective of the present work was to analyze the active components of the MPCE and study their anti-obesity effects in vivo.

Mandarin peel is a natural by-product that is mainly produced while processing citrus fruit. This primary waste has mostly been discarded, leading to environmental and economic problems in the food industry [8]. In this context, there have been many efforts to utilize mandarin peel and elucidate its potential as a promising source of natural phenolic compounds indicating various health-benefiting properties, such as antioxidant, anti-inflammatory, anti-obesity, and anti-diabetes [11,35]. Narirutin and hesperidin, the flavanones highly present in citrus fruits, provide a wide range of multifunctional bioactivities [13]. In particular, narirutin has been identified for its widespread pharmaceutical advantages against various diseases, including obesity [13,17]. Narirutin from the peels of various citrus species has shown anti-adipogenic effects by regulating adipocyte differentiation, lipolysis, and lipid metabolism along with protein and mRNA levels of C/EBP, PPAR, and SREBP1c in 3T3-L1 adipocytes [13]. Additionally, bioflavonoids such as narirutin and hesperidin from lemon extract have also revealed consequent modulation of in vitro adipocyte development and lipid accumulation [36]. Moreover, many previous in vivo studies have suggested that the underlying mechanisms of narirutin prevent obesity and metabolic disorders, according to the inhibition of adipogenesis and lipid accumulation, and promote lipolytic enzymes such as AMPK and HSL [37]. Therefore, the contents of narirutin in MPCE were examined, and it was confirmed that it contained 13.795 mg/g, the highest amount among the five flavanones that were investigated (Figure 1). Previous studies have suggested diverse extraction methods for narirutin from citrus peel using microwave-assisted extraction, subcritical water, and ethanol. However, compared with traditional extraction methods, the enzyme-assisted extraction technique to extract flavonoids from citrus peel has the advantages of extractability by degrading cell walls to promote the dissolution of components, thus increasing the yield and efficacy [21,38]. As expected, the present results showed that narirutin in mandarin peel extracted by Celluclast extraction had a higher content compared to the extraction technique mentioned above [18,39]. These findings demonstrate that enzymatic extraction techniques might be more advantageous than other extraction methods to obtain narirutin from mandarin peel and might make it acceptable as a bioactive extract with functional food and nutraceutical potential.

Our findings identified that MPCE treatment significantly reduced the bodyweight gain of HFD-induced mice (Figure 2B). The obese mice administered with MPCE exhibited significant reductions in body weight, liver, and fat mass compared to the mice fed HFD only. The current results showed the anti-obesity effect of MPCE, which regulates body weight by reducing lipid accumulation in the liver and WAT. Consistently, the levels of blood metabolic parameters associated with obesity were measured in the serum. MPCE supplementation significantly downregulated the blood metabolic parameters, including TG, ALT, AST, and leptin levels, in HFD-induced obese mice. These results indicate that MPCE significantly reduced lipid accumulation-induced liver damage and appetite hormonal dysregulation induced by HFD. In brief, MPCE effectively alleviates harmful changes in obesity and related metabolic disorders via the regulation of systemic lipid formation and blood metabolic parameters.

In the development of obesity, the WAT is a crucial organ that modulates energy metabolism and lipid accumulation. Overexpression of a variety of adipogenic-specific factors, including SREBP1c, C/EBPα, PPARγ, and FAS, can accelerate lipid accumulation in the WAT [40]. Therefore, the inhibition of lipid accumulation by regulating these adipogenic-specific factors may be a possible strategy against obesity. Therefore, in the present study, the expression levels of proteins related to adipogenesis and lipogenesis were investigated to determine the mechanisms underlying the inhibitory effect of MPCE on lipid accumulation in the WAT. The results showed that MPCE notably alleviated the expression levels of C/EBPα, PPARγ, SREBP1c, and FAS, which are responsible for adipogenesis and lipid synthesis. Various studies reported that the enzyme and ethanol extract from citrus peel inhibited adipogenesis and lipid accumulation by regulating the expression levels of the factors mentioned above in 3T3-L1 adipocytes and C57BL/6 mice, respectively [41,42]. Moreover, our previous study demonstrated that MPCE significantly inhibited adipose differentiation by downregulating PPARγ, C/EBPα, and SREBP1c in 3T3-L1 adipocytes [23]. Our present study also identified the effects of MPCE against obese conditions in mammalian models as a follow-up study to support previous in vitro studies. Taken together, we can conclude that the effect of MPCE on HFD-induced obesity is triggered by inhibiting both adipogenesis and lipogenesis in the WAT of obese mice. AMPK is a serine/threonine protein kinase that regulates cellular energy homeostasis and lipid metabolism. Activation of AMPK plays an important role by activating several key metabolic pathways related to obesity, including the inactivation of ACC and stimulation of HSL, which contribute to fatty acid synthesis inhibition and lipolysis promotion, respectively [43]. AMPK is a common target protein to prevent and treat obesity. Therefore, to elucidate the further molecular mechanism by which MPCE inhibits adipogenesis, the protein levels of phosphorylated AMPK and its substrate, ACC, were measured. We found that treatment with MPCE significantly increased phosphorylation levels of AMPK and ACC in the WAT. In addition, MPCE significantly increased the phosphorylation levels of HSL, which plays an important role in lipolysis, in the WAT. However, in obese conditions, excess free fatty acid (FFA) released from lipolysis can contribute to hyperglycemia and insulin resistance, leading to hypertriglyceridemia [44]. Combined with the effects of MPCE supplementation on the reduction of serum TG levels, we speculated that MPCE has the potential to restore impaired FFA)suppression in HFD-induced obese conditions. Interestingly, many previous studies have revealed both in vitro and in vivo anti-diabetic effects of narirutin, the most abundant flavonoid in MPCE, which has been illustrated in our study. Narirutin from various citrus-derived products reduced carbohydrate digestion and improved hyperglycemia and hyperlipidemia by regulating glucose and lipid metabolism, respectively [13]. Taken together, these results suggest that MPCE could reduce lipogenesis and enhance lipolysis with acceptable regulation of FFA release by enhancing AMPK phosphorylation, thereby preventing HFD-induced obesity and related glucose/lipid dysregulation.

NAFLD is a chronic liver disease commonly found in obese patients, accompanied by various metabolic diseases. The obesity-related excessive lipid accumulation in hepatocytes leads to severe hepatic failure, such as hepatitis and fibrosis [45]. Thus, the present study investigated the effect of MPCE on hepatic lipid accumulation and related mechanisms in HFD-induced obese mice. MPCE significantly decreased HFD-induced hepatic lipid accumulation in the livers of obese mice. Consistently, the results showed that MPCE treatment downregulated the expression levels of lipogenic-specific promoters, such as SREBP1c and FAS, and upregulated AMPK and ACC phosphorylation in the liver of HFD-induced obese mice. These results demonstrate that MPCE could both attenuate HFD-induced obesity and prevent obesity-related hepatic diseases by inhibiting hepatic lipid accumulation via the downregulation of lipogenesis and the upregulation of lipolysis.

In conclusion, the present study demonstrated that MPCE obtained from enzymatic extraction contained a considerable amount of narirutin compared with other extraction methods. MPCE also exhibited a potent inhibitory effect on adipogenesis and lipid accumulation in HFD-induced obese mice. Therefore, the enzymatic extraction of mandarin peel is an effective process to make mandarin peel-derived products more acceptable as health foods and nutraceuticals. However, further in-depth studies about energy expenditure and glucose metabolism are warranted to clarify the detailed underlying mechanisms of HFD and MPCE on obesity-related metabolic disorders and glucose metabolism. In summary, mandarin peel Celluclast extract could be utilized as a potential natural curative agent to ameliorate obesity and obesity-induced related metabolic disorders.

## 4. Materials and Methods

### 4.1. Materials and Reagents

Samples of Mandarin (*Citrus unshiu*) peel were kindly provided by ILHAE Co., Republic of Korea. The kit for measuring levels of triglyceride (TG) (#10010303) was purchased from Cayman Chemical (Ann Arbor, MI, USA). The kits for measuring levels of alanine aminotransferase (ALT) (#CSB-E16539m), aspartate aminotransferase (AST) (#CSB-E12649m), and leptin (#CSB-E04650m) were purchased from CUSABIO (Cusabio Technology, Houston, TX, USA). Antibodies against SREBP1c (#sc-366), GAPDH (#sc-365062), and β-actin (#sc-47778) were purchased from Santa Cruz Biotechnology (Santa Cruz, CA, USA). Antibodies against PPARγ (#2443), C/EBPα (#2295), FAS (#3180), ACC (#3676), p-ACC (#11818), AMPKα (#2532), p-AMPKα (#2531), HSL (#4107), and p-HSL (#4126) were purchased from Cell Signaling Technology (Danvers, MA, USA). All other chemicals and reagents used in this study were of analytical grade.

### 4.2. Extraction and Chemical Composition Analysis of MPCE

Mandarin peel Celluclast extract (MPCE) was extracted using a previously reported method [46]. In brief, dried Mandarin peel powder (10 g) was mixed with 1 L of distilled water and 100 μL of Celluclast enzyme (Novo Nordisk, Bagsvaerd, Denmark). This mixture was incubated at 50 °C for 24 h in a shaking incubator. Digestive material was then boiled at 100 °C for 10 min for enzyme inactivation. The supernatant was separated from unhydrolyzed residues by centrifugation at 3000× *g* for 20 min. Finally, the enzymatic extract was adjusted to pH 7.0 and then lyophilized. The lyophilized sample was used as an MPCE sample.

For analysis of flavonoid compositions, the sample was prepared by dissolving 100 mg of MPCE in 10 mL of 80% methanol. After incubation at 30 °C for 3 h, the reaction mixtures were filtered through a 0.45 μm pore filter. The sample (MPCE) and five flavonoid standards (narirutin, naringin, hesperidin, naringenin, and hesperetin) were analyzed using an HPLC system (1260 Infinity, Agilent, Santa Clara, CA, USA) with a reverse-phase column (TSKgel ODS-100V, Tosoh, Tokyo, Japan). For elution of the constituents, two solvents were used as follows: solvent A, 0.1% H_3_PO_4_ in water; solvent B, 0.1% H_3_PO_4_ in acetonitrile; and the eluent was monitored using a diode array detector at 285 nm.

### 4.3. Animal Experiments

Four-week-old male C57BL/6 mice were purchased from Joongah Bio (Suwon, Republic of Korea) and maintained under standard conditions with a 12 h/12 h light/dark cycle in a temperature and humidity-controlled facility. These mice had free access to commercial chow and water. After acclimation to the laboratory environment for one week, mice were randomly divided into the (1) high-fat diet group (HFD, Research Diets Inc., rodent diet containing 60% kcal from fat and 20% kcal from both carbohydrate and protein, 5.21 kcal/g, D12492), (2) low-dose MPCE-administered group (HFD + MPCE (LD)), and (3) high-dose MPCE-administered group (HFD + MPCE (HD)) at *n* = 6 per group. The MPCE was dissolved in distilled water (DW). The mice were administered orally with MPCE at a dose of 125 mg/kg bw (HFD + MPCE (LD)) or 200 mg/kg bw (HFD + MPCE (HD)) once daily for 11 weeks, using oral feeding needles. The mice in other groups were administered an equal volume of DW. The body weights of the mice were recorded weekly during the feeding period. After the last administration, all mice were fasted for 12 h and anesthetized by inhaling ether. Blood, liver, and epididymal white adipose tissue (WAT) were carefully collected and stored at −80 °C for future analysis. 

### 4.4. Blood Parameter Analysis

Blood was collected after fasting for 12 h before dissection, and serum separation was performed by centrifugation at 3000× *g* at 4 °C for 10 min. The TG, ALT, AST, and leptin levels in serum were measured using commercial enzyme-linked immunosorbent assay (ELISA) kits in accordance with the manufacturer’s instructions.

### 4.5. Histological Analysis

The liver tissues and epididymal WAT were fixed with a 10% formalin solution and embedded in paraffin. Sections were stained with hematoxylin and eosin (H&E). After alcohol dehydration, stained areas were photographed at a magnification of 200× using a light microscope (Flexcam C3, Leica Microsystems, Wetzlar, Germany).

### 4.6. Western Blot Analysis

The liver tissues and epididymal WAT were homogenized in cell extraction buffer (Invitrogen, Carlsbad, CA, USA) supplemented with PMSF and a protease inhibitor cocktail (Invitrogen, CA, USA). Total protein concentrations were determined. The Western blot analysis was performed according to the method in a previous study [47]. In brief, sodium dodecyl sulfate polyacrylamide gel electrophoresis (SDS-PAGE) was performed to separate proteins and then transferred to a nitrocellulose membrane. The membrane was blocked in 5% non-fat dry milk in TBS-T for 2 h and then incubated with primary antibodies (dilution of 1:1000) at 4 °C overnight. After washing, the membrane was incubated with secondary antibodies (dilution of 1:3000) at room temperature for 1.5 h. The chemiluminescent detection was completed using ECL Western Blotting Reagents, and protein bands were quantified with ImageJ software version 1.53e.

### 4.7. Statistical Analysis

Data are expressed as the mean ± standard deviation (SD). All experiments were performed in triplicate. Significant differences were determined by one-way analysis of variance (ANOVA), complemented by Duncan’s multiple range test. A statistically significant difference was considered at *p* < 0.05.

## Figures and Tables

**Table 1:** Effect of MPCE on HFD-induced obesity-related blood biological parameters in obese mice.

Parameter	Group		
	HFD	HFD + MPCE (LD)	HFD + MPCE (HD)
TG (mg/dL)	343.42 ± 97.99	284.02 ± 19.17	187.01 ± 32.29 **
AST (mU/mL)	81.51 ± 22.25	30.07 ± 7.88 **	38.12 ± 9.45 **
ALT (U/mL)	81.01 ± 11.56	35.16 ± 4.78 **	43.71 ± 2.18 **
Leptin (ng/mL)	52.43 ± 8.95	40.67 ± 5.15 *	37.98 ± 0.89 **

Data are expressed as the mean ± standard deviation (*n* = 6). * *p* < 0.05, ** *p* < 0.01, indicate significant differences compared with the only HFD group. TG, triglyceride; AST, aspartate aminotransaminase; ALT, alanine aminotransferase.

## Data Availability

All data analyzed during this study are included in this article.
